# Global Analysis of Gene Expression Profiles Provides Novel Insights into the Development and Evolution of the Large Crustacean *Eriocheir sinensis*

**DOI:** 10.1016/j.gpb.2019.01.006

**Published:** 2020-12-18

**Authors:** Jun Wang, Xiaowen Chen, Funan He, Xiao Song, Shu Huang, Wucheng Yue, Yipei Chen, Zhixi Su, Chenghui Wang

**Affiliations:** 1 Key Laboratory of Freshwater Aquatic Genetic Resources, Ministry of Agriculture, Shanghai 201306, China; 2 National Demonstration Center for Experimental Fisheries Science Education, Shanghai Ocean University, Shanghai 201306, China; 3 Shanghai Engineering Research Center of Aquaculture, Shanghai 201306, China; 4 State Key Laboratory of Genetic Engineering, Collaborative Innovation Center of Genetics and Development, School of Life Sciences, Fudan University, Shanghai 200438, China; 5 Department of Thoracic Surgery, Shanghai Pulmonary Hospital, Tongji University, Shanghai 200433, China; 6 Ministry of Education Key Laboratory of Contemporary Anthropology, School of Life Sciences, Fudan University, Shanghai 200438, China

**Keywords:** Developmental transcriptome, Metamorphosis, Evolution, Ecology

## Abstract

Chinese mitten crab (*Eriocheir sinensis*) is an important aquaculture species in Crustacea. Functional analysis, although essential, has been hindered due to the lack of sufficient genomic or transcriptomic resources. In this study, transcriptome sequencing was conducted on 59 samples representing diverse developmental stages (fertilized eggs, zoea, megalopa, three sub-stages of larvae, juvenile crabs, and adult crabs) and different tissues (eyestalk, hepatopancreas, and muscle from juvenile crabs, and eyestalk, hepatopancreas, muscle, heart, stomach, gill, thoracic ganglia, intestine, ovary, and testis from adult crabs) of *E. sinensis*. A comprehensive reference transcriptome was assembled, including 19,023 protein-coding genes. Hierarchical clustering based on 128 differentially expressed cuticle-related genes revealed two distinct expression patterns during the early larval developmental stages, demonstrating the distinct roles of these genes in “crab-like” cuticle formation during metamorphosis and cuticle calcification after molting*.* Phylogenetic analysis of 1406 one-to-one orthologous gene families identified from seven arthropod species and *Caenorhabditis elegans* strongly supported the hypothesis that Malacostraca and Branchiopoda do not form a monophyletic group. Furthermore, Branchiopoda is more phylogenetically closely related to Hexapoda, and the clade of Hexapoda and Branchiopoda and the clade of Malacostraca belong to the Pancrustacea. This study offers a high-quality transcriptome resource for *E. sinensis* and demonstrates the evolutionary relationships of major arthropod groups. The differentially expressed genes identified in this study facilitate further investigation of the cuticle-related gene expression networks which are likely associated with “crab-like” cuticle formation during metamorphosis and cuticle calcification after molting.

## Introduction

Crustacea subphylum, one of the largest groups in the Arthropod phylum, consists of Branchiopoda, Remipedia, Cephalocarida, Maxillopoda, Ostracoda, and Malacostraca [1]. This group exhibits morphological and ecological species diversity. Most of the species in this group inhabit marine, freshwater or humid environments, although there are also some terrestrial species [2]. Several unique biological processes occur in most Crustacea species, including molting, regeneration, metamorphosis, and migration, rendering these species an appropriate invertebrate model system for developmental and functional genomic studies [3], [4], [5], [6], [7], [8], [9], [10]. A deep understanding of the phylogenetic relationships among major Arthropod groups, especially the morphologically diverse Crustacea, is essential for resolving the animal tree of life [11], [12]. Conflicting hypotheses regarding the relative phylogenetic relationships among Malacostraca, Branchiopoda, and Insecta limit the understanding of these relationships, and the resolution of phylogenetic relationships in the Pancrustacea clade of Arthropoda remains a problem [12], [13], [14], [15]. In addition, many Crustacea species, such as crabs, lobsters, and shrimps, are of high aquacultural importance [16], [17]. However, functional and evolutionary studies in Crustacea have been sparse, because there are less assembled reference genomes and additional relevant genome-wide resources compared with those for other Arthropod groups (such as the Hexapoda subphylum) [18].

At present, next-generation sequencing (NGS) technology has developed rapidly, which has led to the assembly and research of high-quality genomes in numerous species [19], [20], [21]. However, only three species in Crustacea possess available draft genome sequences: water flea (*Daphnia pulex*), *Parhyale hawaiensis*, and Chinese mitten crab (*Eriocheir sinensis*) [6], [8], [10]. Given the high rates of heterozygosity and repeats in Crustacea genomes, it is a substantial challenge to generate high-quality genomes of these species [22], [23]. All three of the assembled Crustacea genomes require large improvements in assembly and annotation [6], [8], [10]. Considering that high-quality transcriptome resources have played crucial roles in functional and evolutionary studies in several species without good-quality reference genomes [24], [25], [26], [27], [28], [29], a genome-wide high-quality reference transcriptome may be an alternative option for comparative studies on Crustacea species.

**Figure 1:**
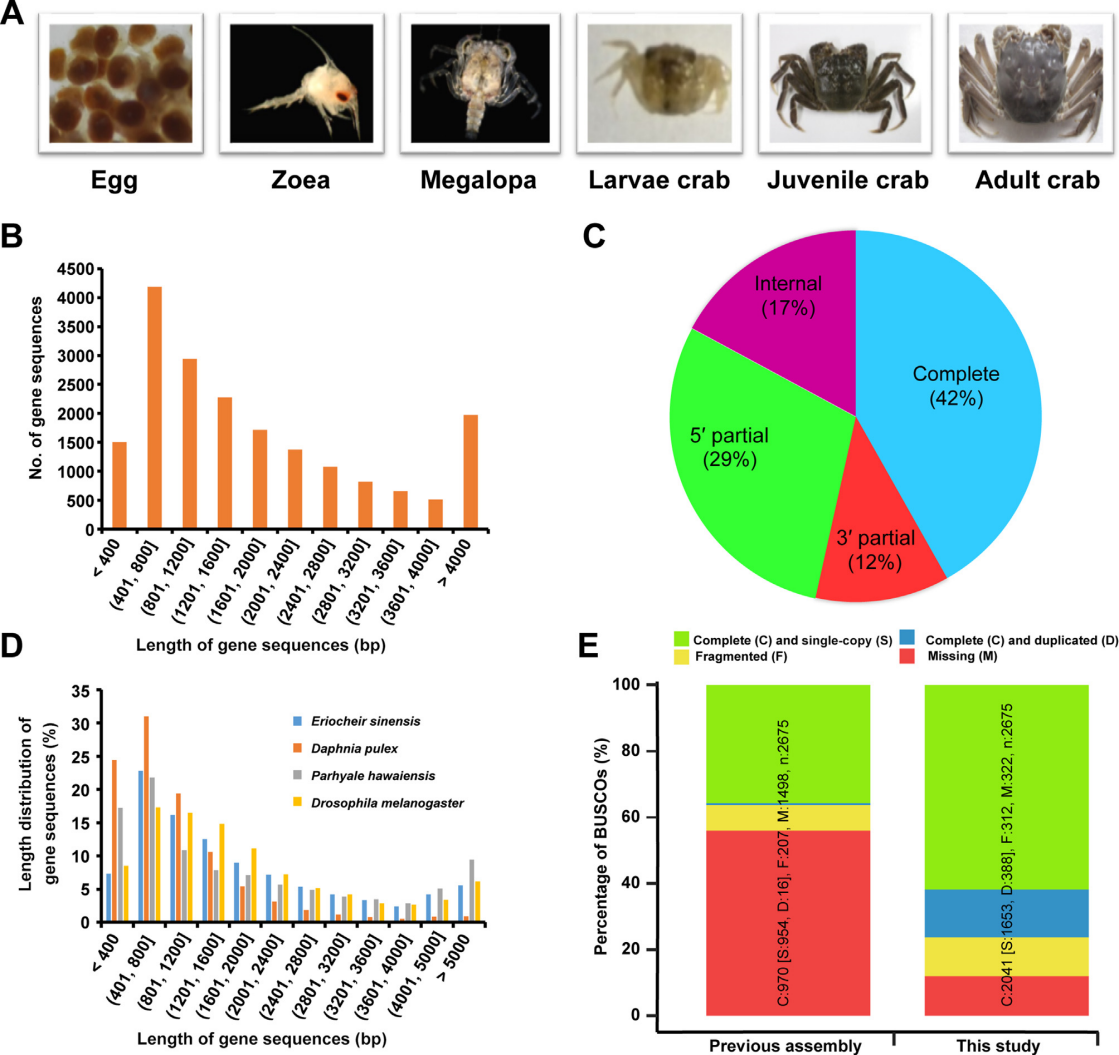
**Developmental stages of *E. sinensis* and transcriptome assembly and annotation assessment** **A.** Six main developmental stages of *E. sinensis* investigated in this study. **B.** Length distribution of predicted coding sequences (CDSs) of *E. sinensis* transcriptome. **C.** Statistics on the completeness of CDSs. Complete, sequences with both the start and stop codons and can be translated to complete protein sequences; 5′ partial, sequences miss a start codon and can be translated to the very 5′ end; 3′ partial, sequences miss a stop codon and can be translated to the very 3′ end; internal, sequences miss both the start and stop codons and can be translated from the first to the last base pair in the sequence. **D.** Length distribution of predicted CDSs among *E. sinensis*, *Daphnia pulex*, *Parhyale hawaiensis*, and *Drosophila melanogaster*. **E.** Comparison of transcriptome completeness between this study and previous study based on the arthropod dataset using BUSCO v1.2 software.

Compared with *D. pulex* and *P. hawaiensis*, *E. sinensis* is a representative farmed large Crustacea species with high nutritional and economic values and is widely aquacultured in China [30]. It is also considered as an invasive species in other countries, because it causes severe ecological destruction due to its burrowing activity [31], [32]. Characteristic biological processes of Crustacea species are present in *E. sinensis*, including 1) transition from swimming to crawling (metamorphosis) during the larval developmental stage, 2) migration from seawater to freshwater for growth and migration from freshwater to seawater for breeding, 3) limb regeneration, and 4) periodic molting during the entire developmental process before sex mature [7], [10], [30]. Although transcriptome studies on several tissues and developmental stages of *E. sinensis* have been conducted [7], [33], [34], [35], [36], there lacks a comprehensive transcriptomic resource across different developmental stages for *E. sinensis*. Due to the limited genomic resources available for *E. sinensis*, the mechanisms underlying the metamorphosis, migration, molting, and regeneration of this species as well as the ecological management solutions and the evolutionary relationships of arthropods remain unclear. Therefore, improving the assembly and annotation of *E. sinensis* transcriptome is necessary. In this study, RNA-seq was conducted using samples from six main developmental stages covering the total life history and different tissues of *E. sinensis* (Figure 1A). A comprehensive transcriptome was assembled to improve the assembly and annotation of *E. sinensis*. The metamorphosis process in *E. sinensis* was investigated and the evolutionary relationships of *E. sinensis* with other arthropods were also depicted.

## Results

### Transcriptome assembly and annotation

A total of 1,205,288,037 paired-end reads (2 × 150 bp read length) were obtained from 59 samples collected at different developmental stages [fertilized eggs, zoea, megalopa, larvae (stage I, stage III, and stage V), one-year-old juvenile crabs, and two-year-old sexually mature adult crabs], or from different tissues (eyestalk, hepatopancreas, and muscle of juvenile crabs, and eyestalk, hepatopancreas, muscle, heart, stomach, gill, thoracic ganglia, intestine, ovary, and testis of adult crabs) (Table 1, Table S1). A total of 934,336,878 paired-end clean reads (~ 280 Gb) were generated after quality trimming, and used for transcriptome assembly, resulting in 259,639 assembled transcripts. After removal of redundant transcripts and transcripts with kilobase per transcript per million mapped reads (FPKM) < 0.5, 66,713 transcripts were retained and used as a reference transcriptome for downstream analysis (Table 1; Figure S1). The N50 length and the average length of the filtered transcripts were 2323 bp and 1440 bp, respectively (Table 1; Figure S1). Mapping the clean reads from each sample to the assembled reference transcriptome showed a mapping proportion of over 90% (Table 1; Figures S1 and S2).

**Table 1:** **Summary of assembled *E. sinensis* transcriptome**

**Type**	**Information**	**Value**
Sequencing	Raw reads (paired-end)	1,205,288,037
	Clean reads (paired-end)	934,336,878
	Total clean nucleotides (bp)	∼2.8 × 10^11^

Assembly	No. of transcripts	66,713
	No. of N50 transcripts	11,840
	N50 length (bp)	2323
	Mean length of transcripts (bp)	1440
	Largest length of transcripts (bp)	17,681
	Mapping rate	98.02%

Annotation	NCBI-NR	33,555
	Uniprot	26,783
	GO	26,984
	KEGG	20,580

*Note*: GO, Gene Ontology; KEGG, Kyoto Encyclopedia of Genes and Genomes.

Using TransDecoder software, we predicted 33,820 coding sequences (CDSs) from the assembled transcriptome. The sequences were derived from 19,023 protein-coding genes, the average nucleic acid length was 1940 bp (Figure 1B; Table S2), and more than 40% of the predicted CDSs were structurally completed (start and stop codons were both presents) (Figure 1C). The CDS length distribution of *E. sinensis* showed a similar pattern to fruit fly (*Drosophila melanogaster*), which possesses a more complete genome than water flea (*D. pulex*) (Figure 1D). Our assembly recovered nearly 88% of the conserved orthologous genes of the arthropod dataset included in the Benchmarking Universal Single-Copy Orthologs (BUSCO) analysis (Figure 1E). Compared with previous gene models predicted from the draft genome of *E. sinensis*, more annotated gene models, longer gene length, and more completeness of gene models were identified in this study through BUSCO analysis based on the single-copy orthologs using the arthropod dataset (Figure 1; Table S2).

**Supplementary Figure S3:**
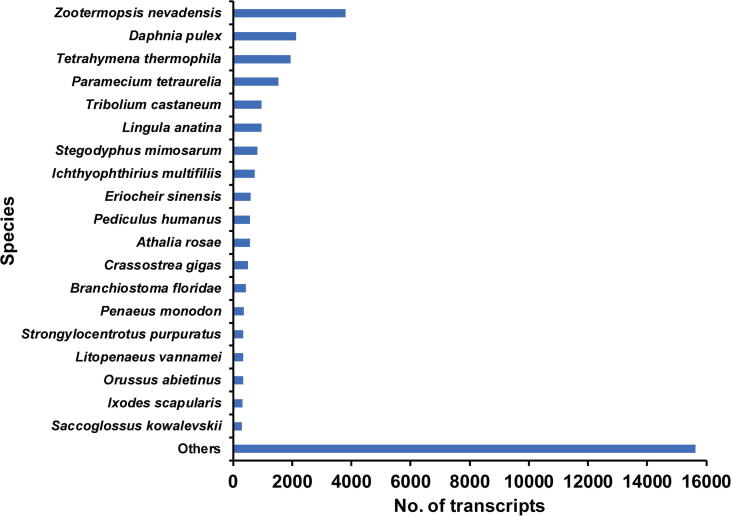
**Species distribution of top-blast hits in the assembled referencetranscriptome**

**Supplementary Figure S4:**
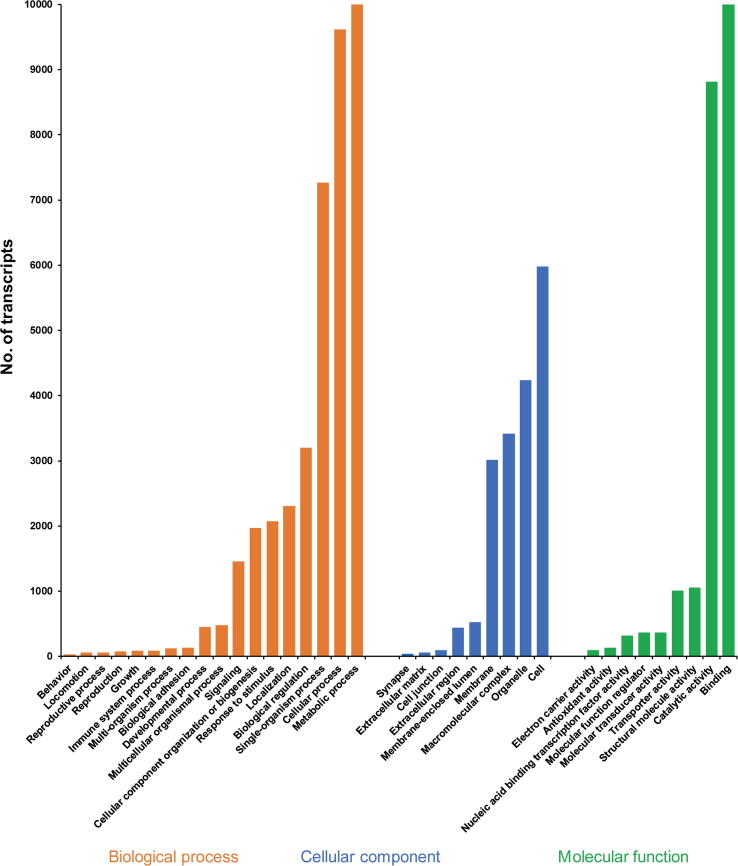
**Functional classification of the transcriptome assembled from *E. sinensis* based on three main Gene Ontology (GO)categories**

Based on BLASTX annotation using NCBI-NR and UniProt protein databases, 33,555 (50.3%) and 26,783 (40.1%) of the transcripts could be annotated, respectively (Table 1). In total, 12,015 protein-coding genes could be annotated by the above indicated protein databases in this study. Most of the top BLAST hits were for *Zootermopsis nevadensis*, followed by *D. pulex* (Figure S3). After Gene Ontology (GO) mapping, 26,984 transcripts were assigned to at least one GO term using Blast2GO software (Figure S4). Meanwhile, a total of 20,580 transcripts were assigned to 124 Kyoto Encyclopedia of Genes and Genomes (KEGG) pathways and 404 corresponding enzymes (Table S3). 645 protein sequences were predicted to be transcription factors (TFs) using HMMER software with the PFAM-A database (Table S4). A total of 155 assembled transcripts showed significant sequence similarity to 44 non-coding RNAs of *D. melanogaster* (Table S5).

### Gene expression and differential expression

In this study, genes exhibiting FPKM ≥ 1 in all biological replicates were defined as expressed. After calibration, the number of expressed genes among different developmental stages ranged from 10,567 to 16,163, and the number of annotated genes ranged from 6471 to 8817 (Table S6). Among the examined developmental stages, the fertilized eggs contained the fewest expressed genes, whereas the sexually mature adult crabs displayed the largest number of expressed genes (Table S6). In the various tissues of the sexually mature adult crabs, the number of genes expressed in a given tissue ranged from 7454 to 10,517, with the gill and eyestalk presenting the largest number of expressed genes and the intestine and muscle containing the fewest expressed genes (Table S6).

The number of differentially expressed genes (DEGs) identified in this study varied among the pairwise comparisons (Table S7). Only one DEG was identified between stage III and stage V larvae, while 6774 DEGs were identified between the juvenile eyestalk and adult hepatopancreas tissues (Table S7). Among the examined tissues of adult crabs, 303, 108, 951, 21, 44, 311, and 70 DEGs were identified as being upregulated in the hepatopancreas, eyestalk, gill, muscle, heart, ovary, and testis, respectively, compared with each of the other studied tissues in adult crabs. GO enrichment analysis indicated that highly expressed genes in the eyestalk were enriched in photoreceptor activity (GO:0009881), neuropeptide hormone activity (GO:0005184), phototransduction (GO:0007602), and G-protein coupled receptor signaling pathway (GO:0007186) (Table S8). In gills, highly expressed genes were enriched in ammonium transmembrane transport (GO:0072488) and organic cation transport (GO:0015695) (Table S8). In the heart tissue, highly expressed genes were enriched in neuron fate commitment (GO:0048663), blood vessel morphogenesis (GO:0048514), defense response regulation (GO:0031347), and heart morphogenesis (GO:0003007) (Table S8). Genes associated with vitelline membrane formation (GO:0030704), lipid transport (GO:0006869), and pigment metabolic process (GO:0042440) were enriched in hepatopancreas (Table S8). Genes related to stress response (GO:0006950) were identified as highly expressed in muscle (Table S8). In the ovary, highly expressed genes were enriched in cell adhesion (GO:0007155), cilium morphogenesis (GO:0060271), and receptor-mediated endocytosis (GO:0006898). By contrast, genes related to negative regulation of immune system process (GO:0002683), regulation of cell motility (GO:2000145), RNA-dependent DNA replication (GO:0006278), and protein import into nucleus (GO:0006606) were enriched in testis (Table S8).

**Supplementary Figure S5:**
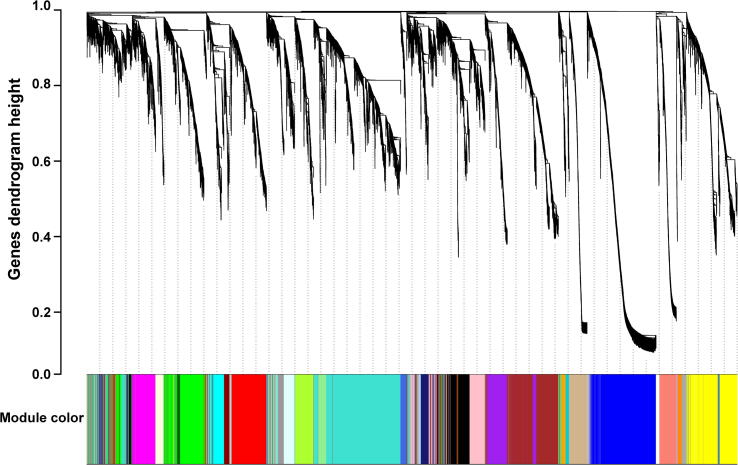
**Linkage clustering tree (dendrogram) based on topological overlap distance in gene expression profile of all genes, together with assigned modulecolors**

Among all genes in the constructed gene coexpression network, 18,787 genes were assigned to 32 modules (Figure S5; Table S9). The number of genes in different modules ranged from 36 to 2878. GO enrichment analysis indicated different modules associated with specific biological functions (Figure S5; Table S9). Genes in the turquoise module may be associated with vitelline membrane formation; those in the brown module may be involved in embryonic development; the red module is strongly associated with visual development; the cyan module is associated with nervous system development to a certain extent. Genes associated with the chitin metabolic process and steroid hormone-mediated signaling pathways were enriched in the yellow, dark orange, white, and violet modules (Figure S5; Table S9).

### Differential expression during larval development

**Figure 2:**
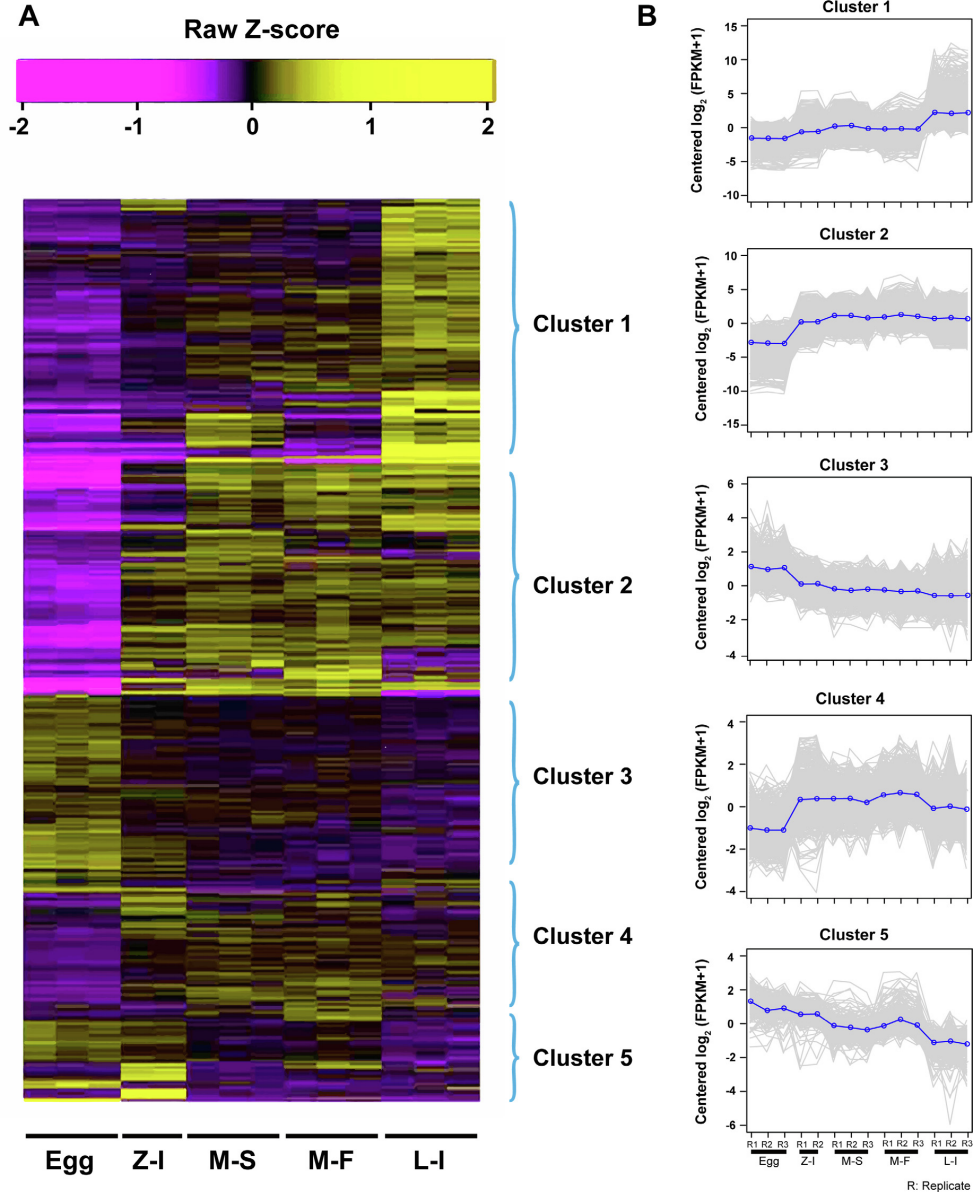
**Expression patterns of DEGs from fertilized eggs to stage I larvae** **A.** Heatmap of DEGs. **B.** Clusters of coexpressed genes. Egg, fertilized egg stage; Z-I, zoea stage I; M-S, megalopa in seawater stage; M-F, megalopa in freshwater stage; L-I: larval stage I. DEG, differentially expressed gene.

A total of 8281 DEGs were identified from fertilized eggs to larval developmental stages [fold change > 2^2^, *P* < 0.001 for false discovery rate (FDR)]. Among these DEGs, 3596 were identified between the egg and zoea stages, 1427 between zoea and megalopa in seawater, 843 between megalopa in seawater and megalopa in freshwater, and 1773 between megalopa in freshwater and stage I larvae (Figure 2A). Five clusters were clearly defined based on the expression values (FPKM) of DEGs during the larval developmental process (Figure 2B). Genes in Cluster 1, which were highly expressed in stage I larvae, were enriched in the structural constituents of cuticle (GO:0042302), growth factor activity (GO:0008083), and chitin metabolic process (GO:0006030) (Figure 2B, Cluster 1). Genes in Cluster 2, which started to be highly expressed at the zoea stage, were enriched in the nervous system development (GO:0007218 and GO:0006836), visual perception (GO:0007601), fatty acid biosynthetic process (GO:0006633), and carbohydrate metabolic process (GO:0005975) (Figure 2B, Cluster 2). Genes in Cluster 3, which were highly expressed in fertilized eggs, were enriched in the DNA replication process (GO:0006260), DNA repair (GO:0006281), and DNA replication initiation (GO:0006270) (Figure 2B, Cluster 3). Genes in Cluster 4, which were highly expressed at the zoea and megalopa stages, were enriched in the oxidation–reduction process (GO:0055114) and G-protein coupled receptor signaling pathway (GO:0007186) (Figure 2B, Cluster 4). In Cluster 5, genes were highly expressed at the egg and zoea stages, and enriched in the DNA-binding (GO:0003677) and regulation of transcription and DNA-templated (GO:0006355) (Figure 2B, Cluster 5).

**Figure 3:**
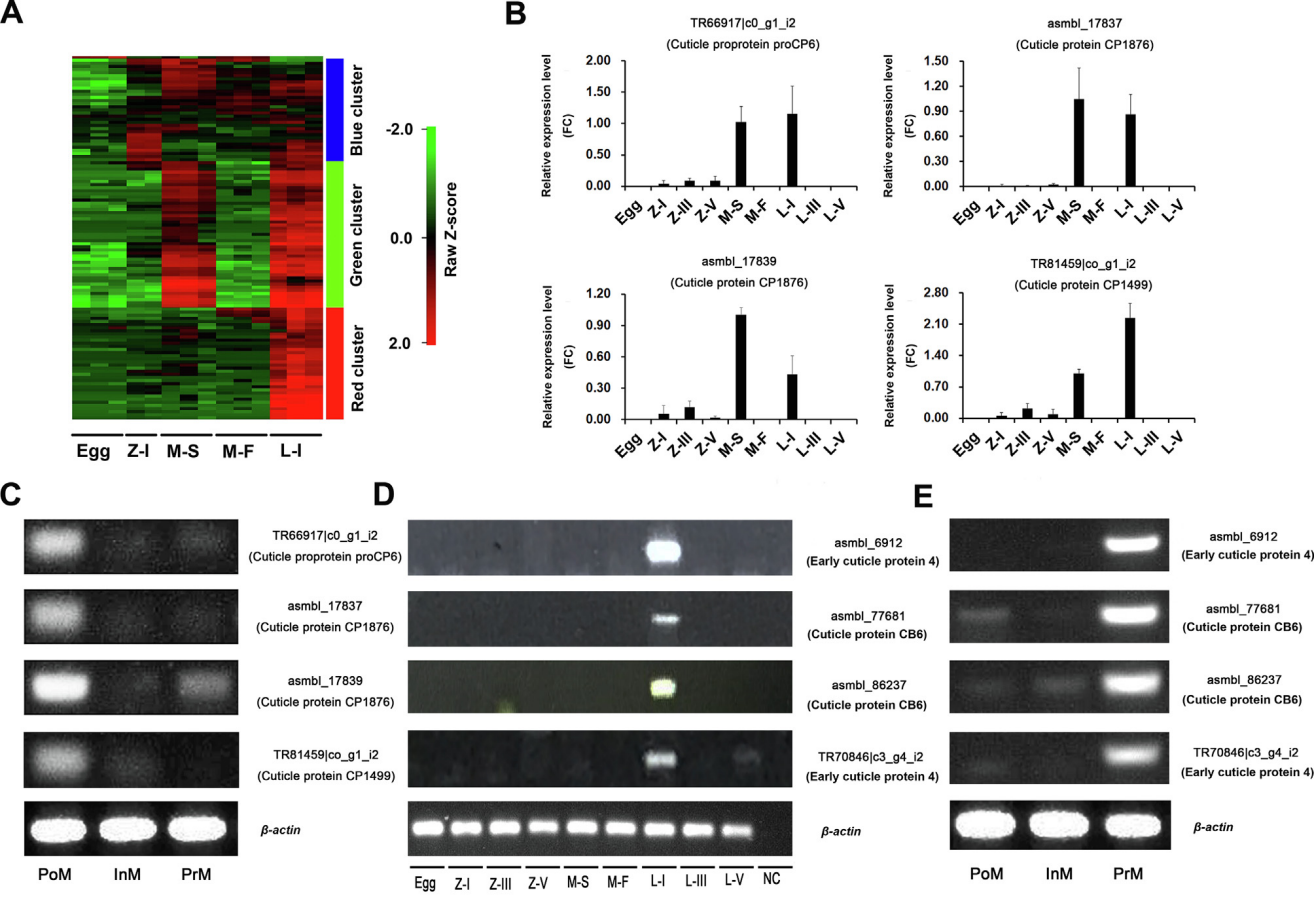
**Heatmap of cuticle-related gene expression and validation at different larval developmental and molting stages** **A.** Heatmap of 128 differentially expressed cuticle-related genes (GO:0042302) during larval developmental stages. Three biological replicates were sampled for egg, megalopa in seawater, megalopa in freshwater, and larval stage I. Two biological replicates for zoea stage I. **B.** qRT-PCR analysis of the relative expression levels of four genes (TR66917|c0_g1_i2, asmbl_17837, asmbl_17839, and TR81459|c0_g1_i2) at different developmental stages. **C.** Semi-quantitative PCR results for the expression levels of four genes (TR66917|c0_g1_i2, asmbl_17837, asmbl_17839, and TR81459|c0_g1_i2) at different molting stages. **D.** Semi-quantitative PCR results for the expression levels of four genes (asmbl_6912, asmbl_77681, asmbl_86237, and TR70846|c3_g4_i2) at different developmental stages. **E.** Semi-quantitative PCR results for the expression levels of four genes (asmbl_6912, asmbl_77681, asmbl_86237, and TR70846|c3_g4_i2) at different molting stages. Z-III, zoea stage III; Z-V, zoea stage V; L-III, larval stage III; L-V, larval stage V; PoM, postmolt stage; InM, intermolt stage; PrM, premolt stage; NC, negative control. *β-actin* was used as a loading control.

Genes related to the structural constituents of cuticle (GO:0042302) and chitin metabolic process (GO:0006030) showed dynamic expression during the larval development stages (from fertilized eggs to stage I larvae). 128 cuticle-related genes were extracted to depict the “cuticle” gene expression pattern. A gene expression heatmap of these genes was generated based on hierarchical clustering, and three clusters were clearly identified (Figure 3A). 66 cuticle-related genes were highly expressed in the megalopa in seawater and stage I larvae, including genes encoding cuticle proprotein proCP6 (ABR27688.1), cuticle protein CP1876 (P81584), and cuticle protein CP1499 (P81583) (green cluster, Figure 3A; Table S10). The high expression levels of these genes were further confirmed by qRT-PCR (*P* < 0.05) (Figure 3B). Meanwhile, these genes were highly expressed at the postmolt stage of *E. sinensis* during the molting cycle (Figure 3C). We identified 45 genes that were highly or specifically expressed in stage I larvae (red cluster, Figure 3A). For instance, genes encoding early cuticle protein 4 and cuticle protein CB6 were almost exclusively expressed at this stage (Figure 3D; Table S10). Additionally, these genes were highly expressed at the premolt stage of *E. sinensis* during the molting cycle (Figure 3E).

### Comparative genomic analyses

**Figure 4:**
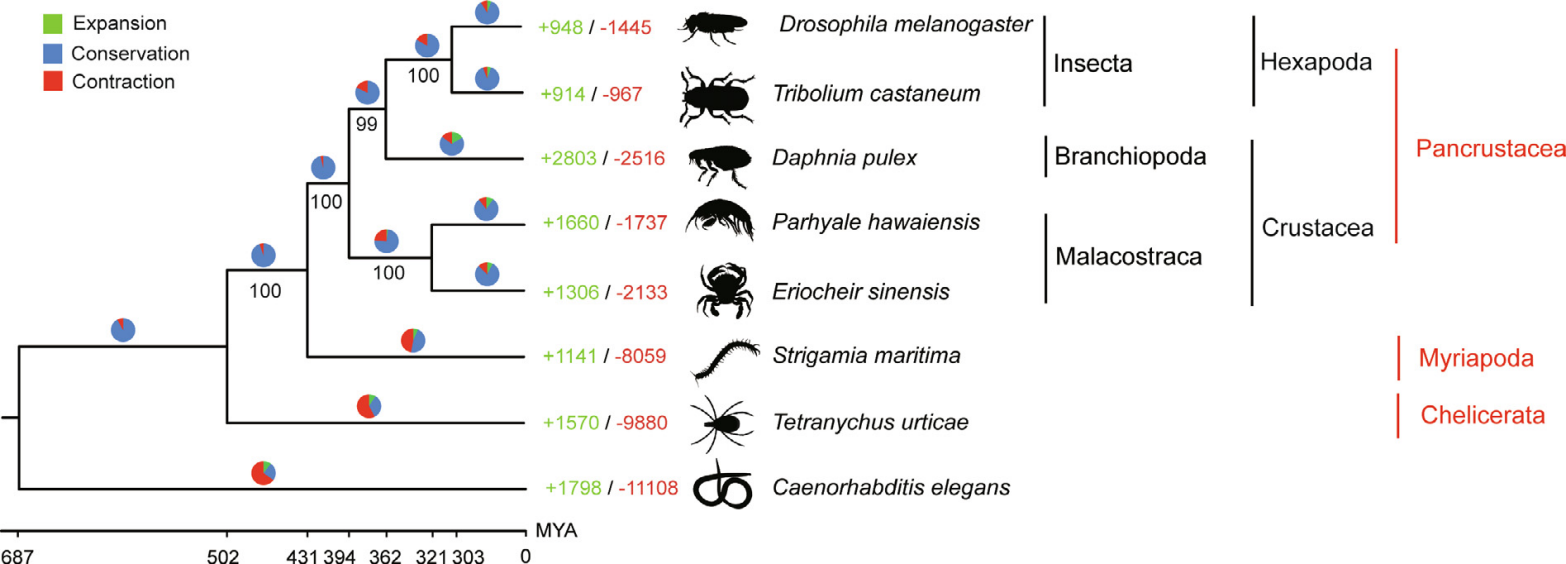
**Phylogenetic relationship and gene family expansion/contraction of *E. sinensis* and other model Arthropod species** The phylogenetic tree was constructed based on the amino acid sequences of 1406 one-to-one orthologous genes in eight selected species. The expansion and contraction of gene families in the eight species were detected with CAFE, where the numbers represent the number of expanded (green) or contracted (red) gene families. The pie chart is proportional to the total number of gene families that have been expanded (green), contracted (red), and conserved (blue). MCMCTree was used to predict the divergence time (in million years) at each node (denoted below). CAFE, Computational Analysis of gene Family Evolution. MYA, million years ago.

Based on the gene model comparison of *E. sinensis* with six other arthropods as well as the outgroup of *Caenorhabditis elegans*, 1406 one-to-one orthologous gene families were identified in the eight species (Table S11). We constructed a phylogenetic tree using RAxML software based on the amino acid sequences of these genes (Figure 4). Strong bootstrap support indicated that flour beetle (*Tribolium castaneum*), fruit fly (*D. melanogaster*), and water flea (*D. pulex*) form a clade, with the clade of *P. hawaiensis* and *E. sinensis* as a sister group (Figure 4). The MCMCTree suggested that *D. pulex* diverged from the common ancestor of insects approximately 362 million years ago (MYA) [with a 95% confidence interval (CI) of 243–464 MYA] (Figure 4). In addition, the divergence time of the clade of *P. hawaiensis* and *E. sinensis* and the clade of *D. pulex*, *D. melanogaster*, and *T. castaneum* was estimated to be approximately 394 MYA (with a 95% CI of 264–513 MYA).

**Supplementary Figure S6:**
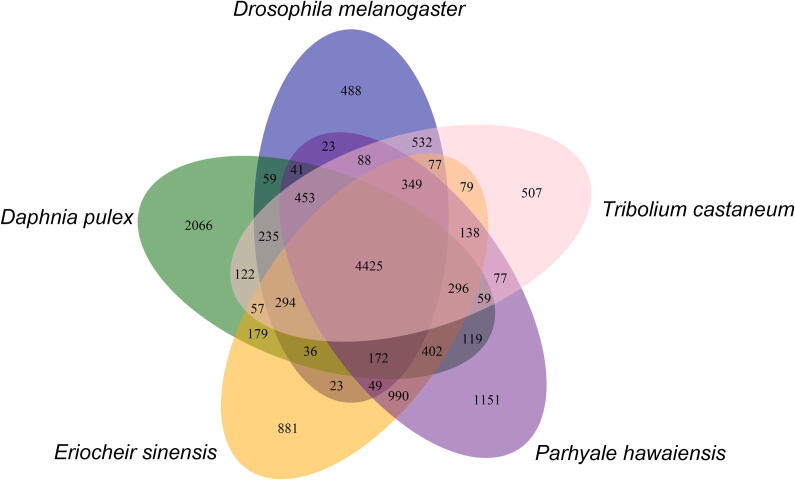
**Venn diagram of shared gene families in five Pancrustacea, including Chinese mitten crab (*Eriocheir sinensis*), amphipod (*Parhyale hawaiensis*), fruit fly (*Drosophila melanogaster*), flour beetle (*Tribolium castaneum*) and water flea (*Daphnia pulex*)** Gene families were estimated by orthoMCL

A total of 17,127 gene families were detected by orthoMCL, among which 1406 were identified as being one-to-one orthologous in all eight species in this study. We identified 4425 gene families that could be detected in all five Pancrustacea species, including *D. pulex*, *T. castaneum*, *D. melanogaster*, *E. sinensis*, and *P. hawaiensis*. We predicted 881 gene families that may have specifically originated from the common ancestor of all five Pancrustacea species (Table S12). Moreover, we identified 881 *E. sinensis*-specific gene families in the five Pancrustacea species (Figure S6) that were significantly enriched in the GO categories of translation and active ammonium transport (Table S13).

**Supplementary Figure S7:**
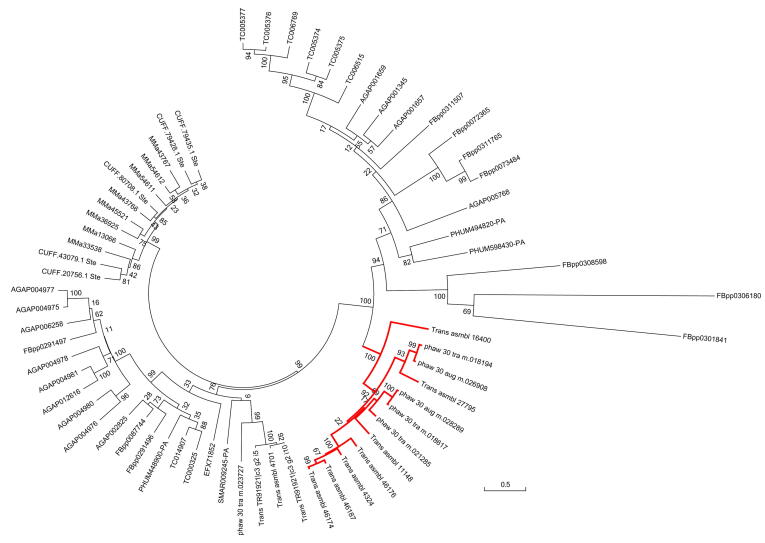
**Maximum-likelihood phylogeny of the hemocyanin superfamily in several representative arthropod genomes** Hemocyanin superfamily proteins were from *Tribolium castaneum, Drosophila melanogaster, Anopheles gambiae, Eriocheir sinensis, Parhyale hawaiensis, Daphnia pulex, Strigamia maritima, Pediculus humanus corporis, Stegodyphus mimosarum*, and *Mesobuthus martensii*. The clade marked with red branches shows the expansion of hemocyanin in Malacostraca. Several partial sequences of crab and *Parhyale hawaiensis* were not included when reconstructing the phylogenetic tree.

Next, we used CAFE (Computational Analysis of gene Family Evolution) software to analyze the expansion and contraction of all gene families detected by orthoMCL. In the *E. sinensis* lineage, 1306 gene families were significantly expanded, and 2133 gene families were significantly contracted (Figure 4). Among the 13 highly expanded gene families observed in *E. sinensis*, two gene families (led684 and led858) presented considerable expansion in the chitin-related protein domains (Chitin_bind_4 and CBM_14 domains) (Table S14). We found that *E. sinensis* contains the largest hemocyanin superfamily among all studied arthropods (Figure S7). Among the 25 annotated hemocyanin proteins in *E. sinensis*, 18 proteins belong to the hemocyanin subfamily, and 7 proteins belong to the prophenoloxidase subfamily. Given the existence of non-overlapping transcript fragments, the total number of predicted hemocyanin superfamily members might be an overestimation.

## Discussion

### Comprehensive reference transcriptome

As far as we know, this study generated the most comprehensive *E. sinensis* transcriptome at a single-base resolution. To broadly sample the transcriptome, we performed paired-end sequencing of poly(A)^+^ RNA in biological duplicates from 59 dissected tissue samples or whole-animal samples from different developmental stages. Compared with other assembled transcriptomes of *E. sinensis*, the reference transcriptome obtained in this study was the most complete, with longer, more annotated, and more structurally complete genes [7], [33], [34], [35], [36] (Figure 1; Table 1). Compared with other Crustacea species with available genomes, such as *D. pulex* and *P. hawaiensis*, our assembled reference transcriptome was also of high quality (Figure 1). The gene expression profiles and DEGs obtained from different developmental stages and tissues of *E. sinensis* are valuable genetic resources for further functional studies in crustaceans. Coexpression analysis results will help us characterize the unannotated gene functions and pave the way for the study of specific biological processes in *E. sinensis* in the future [37], [38]. Our assembled comprehensive transcriptome with detailed annotation information and expression profiles from all examined developmental stages and tissues provides essential genome-wide transcriptomic resources for *E. sinensis*. Furthermore, these data will allow us to explore the mechanisms of unique biological processes in unprecedented detail and solve the mystery of the evolutionary trajectories of Crustacea in future research.

### Biological processes of metamorphosis and molting

Metamorphosis is a widespread life history strategy of animals. However, with the exception of certain model organisms, this process is poorly characterized in Crustacea [39], [40]. The metamorphosis of crustaceans often includes a series of dramatic morphological and physiological changes and usually changes the behavior and habit of the larvae [41]. In *E. sinensis*, morphology changes markedly after “megalopa-to-stage I larvae” metamorphosis, from being “shrimp-like” to “crab-like”, and movement behavior changes from straight to transverse movement [30]. In our study, during the metamorphosis of *E. sinensis*, genes encoding early cuticle protein 4 and cuticle protein CB6 were both specifically expressed in stage I larvae, possibly indicating that these genes are indispensable for “crab-like” cuticle formation during metamorphosis. In *E. sinensis*, the metamorphosis from megalopa to stage I larvae is accompanied by molting. At larval stage I, *E. sinensis* forms a “crab-like” morphology for the first time in its whole life. After larval stage I, the crab remains “crab-like” after every molting cycle. Therefore, these genes may be highly expressed at the premolt stage of each molt, when crabs regenerate their new “crab-like” cuticles. Our PCR results confirmed that genes encoding early cuticle protein 4 and cuticle protein CB6 were highly expressed at the premolt stage of *E. sinensis* (Figure 3E).

The genes encoding cuticle proprotein proCP6, cuticle protein CP1876, and cuticle protein CP1499, which were identified as DEGs, were all upregulated in seawater megalopa and stage I larvae during larval development stages (green cluster, Figure 3A; Figure 3B). Seawater megalopa and stage I larvae were two postmolt stages; therefore, we speculated that these genes may be associated with cuticle calcification after molting. Moreover, our PCR results verified the high expression of these genes at the postmolt stage, during which the cuticles of *E. sinensis* harden within a short period of time. Based on our results, the cuticle-related genes identified in our study may perform at least two main functions: 1) several genes, such as genes encoding early cuticle protein 4 and cuticle protein CB6, are essential for “crab-like” cuticle formation, which may be the genetic basis for the megalopa-to-stage I larvae metamorphosis process; and 2) several genes, such as genes encoding cuticle proprotein proCP6, cuticle protein CP 1876, and cuticle protein CP1499, play vital roles in cuticle calcification after molting. A previous study indicated that a large proportion of transcripts putatively encoding cuticle proteins associated with cuticle formation, calcification, and chitin binding showed dynamic expression throughout the metamorphosis process, which was consistent with our results [42]. Additionally, expanded chitin-related domain family and hemocyanin superfamily were identified in *E. sinensis*; Chitin_bind_4 and CBM_14 in the Pfam domain database are believed to be associated with cuticle formation and calcification in other species, and hemocyanin has been shown to be associated with cuticle formation [43]. The expanded gene families identified in this study indicate that these genes may be vital in megalopa-to-stage I larvae metamorphosis, which will contribute to “crab-like” cuticle formation. As shown in the phylogenetic tree (Figure S7), the hemocyanin subfamily is expanded extensively along the lineage of *E. sinensis* and *P. hawaiensis*, indicating that the expansion of the hemocyanin subfamily may also be associated with the adaptation of Malacostraca to diverse environments at different developmental stages.

### Arthropod phylogenetic relationships

In this study, genome-wide orthologous gene sequences from seven Arthropod species and one outgroup of *C. elegans* were obtained to assess the relationships of arthropods. The evolutionary relationship between Crustacea and Hexapoda has long been controversial [12], [13], [14], [44], [45]. A previous study has classified Crustacea and Hexapoda into the Pancrustacea clade, indicating that terricolous insect species are closely related to the aquicolous Crustacea species [14]. Our results also confirmed the hypothesis that Crustacea are closely related to Hexapoda and should be included in the Pancrustacea clade at the whole-genome level (Figure 4). Additionally, according to our results, Branchiopod and Malacostraca are two independent branches in the phylogenetic tree, and Branchiopod is more phylogenetically closely related to Insecta. The clade of Branchiopoda and Insecta is a sister group of Malacostraca, which demonstrated that Crustacea are a polyphyletic group. Regarding the evolutionary status of Branchiopoda, two available hypotheses consider Branchiopoda together with either Cephalocarida, Remipedia, and Hexapoda [12], [15] or Copepoda, Thecostraca, and Malacostraca [14]. Species in the two groups (Cephalocarida and Remipedia) were not included in this study due to the lack of available genome data; however, our study strongly supports Branchiopoda as a sister group of Hexapoda. Furthermore, our results provide strong evidence supporting recent claims that Malacostraca and Branchiopoda do not form a monophyletic group [8]. The credible phylogenetic results clearly depict the evolutionary relationship of Crustacea and Branchiopoda depending on our assembled high-quality reference transcriptome.

## Conclusion

In conclusion, we assembled and annotated the most comprehensive *E. sinensis* transcriptome. Using the assembled reference transcriptome of *E. sinensis*, we were able to reveal the genetic basis of metamorphosis during larval development and infer arthropod phylogenetic relationships. Our study detected cuticle-related genes likely involved in “crab-like” cuticle formation during metamorphosis and cuticle calcification after molting. Our results strongly support the hypothesis that Crustacea and Hexapoda are closely related and should be included in the Pancrustacea clade. The transcriptomic resources, along with the findings, will play essential roles in further developmental, evolutionary, and aquaculture-related studies in *E. sinensis* and other Crustacea species.

## Materials and methods

### Animal sampling

During the development of *E. sinensis*, fertilized eggs, zoea (stage I, stage III, and stage V), megalopa (megalopa in seawater and in freshwater), and larvae (stage I, stage III, and stage V), as well as different tissues of one-year-old juvenile crabs (eyestalk, hepatopancreas, and muscle) and two-year-old sexually mature adult crabs (eyestalk, hepatopancreas, muscle, heart, stomach, gill, thoracic ganglia, intestine, ovary, and testis), were collected from our aquaculture research base in Shanghai, China (Figure 1A). For the developmental stages before juvenile crabs, mixed whole individuals were collected in three biological replicates, and 10–20 individuals were included in each replicate. However, we collected only two replicates for the zoea I stage for RNA-seq experiment. For the tissues of juvenile and adult crabs, three individual crabs were sampled. All crabs were dissected after anesthesia with ice, and the whole body/tissues were quickly sampled and stored at −80 °C before RNA extraction. Our study and sampling procedures were approved and followed the regulations of the Institutional Animal Care and Use Committee (IACUS) of Shanghai Ocean University (SHOU-DW-2016-004).

### RNA extraction and transcriptome sequencing

RNA was extracted and purified from the collected samples using the RNAiso plus Reagent (Takara, Dalian, China). The concentration and integrity of extracted RNA were measured with NanoDrop 2000 (ThermoFisher Scientific, Shanghai, China) and an Agilent 2100 Bioanalyzer (Agilent, Shanghai, China). A total of >2 μg of RNA with RNA integrity number (RIN) > 8.0 and 1.8 < OD_260/280_ < 2.2 was used for RNA-seq library construction. The sequenced libraries were prepared with Truseq^TM^ RNA sample prep Kit (Illumina, San Diego, CA) for Illumina and the indexed paired-end sequencing libraries were sequenced on the Illumina HiSeq 4000 platform (2 × 150 bp read length).

### Transcriptome assembly and annotation

Raw sequencing reads with low quality were trimmed using the Trimmomatic read trimming tool before assembly [46]. Then, a comprehensive transcriptome database for *E. sinensis* was established using PASA2 software with the following assembly pipeline. First, we conducted *de novo* assembly of a transcriptome using Trinity 2.0.6 software with the assembled contig length above 300 bp [47]. Second, we assembled a transcriptome using Trinity in genome-guide mode with the previous published *E. sinensis* genome as a reference [10]. Finally, we used PASA2 software to merge the two assemblies and obtained a comprehensive reference transcriptome set for *E. sinensis* [48]. To evaluate the representation of RNA-seq reads in the assembly, all raw reads from each developmental stage and tissue were mapped back to the assembled transcriptome to estimate the mapping statistics using bwa-0.17 and SAMtools-0.18 software [49], [50].

NCBI-NR, UniProt, GO, and KEGG databases were used for the functional annotation of the assembled reference transcriptome using BLASTX (E-value < 1E−6). Functional GO assignments and KEGG pathway annotations were performed using BLAST2GO 3.0 software [51]. CDSs were predicted by TransDecoder 2.0.1 software, and the minimal protein sequence length was set to be 100. Completeness information of the predicted CDSs was also recorded. cDNA sequences of *D. pulex* and *D. melanogaster* were downloaded from the Ensembl Metazoa website (http://metazoa.ensembl.org/info/website/ftp/index.html) [52], and cDNA sequence data of *P. hawaiensis* were downloaded from https://figshare.com/articles/supplemental_data_for_Parhyale_hawaniensis_genome/3498104 [8]. For the prediction of functional domains in the protein sequences, all the assembled sequences were blasted against the PFAM-A database using HMMER software [53]. For the prediction of TFs, the curated DNA binding domain list (hidden Markov models) was downloaded from the transcription factor prediction database (release 2.0) (http://www.transcriptionfactor.org) and used as a reference. The completeness of the transcriptome assembly and annotations were evaluated with BUSCO v1.2 software using the arthropod dataset as a reference database, which was downloaded from BUSCO website (http://busco.ezlab.org/v1/) [54]. For the prediction of non-coding RNAs, the assembled transcripts of *E. sinensis* without ORF prediction and functional annotations were used as a query for BLASTN search against (E-value < 1E−10) the non-coding sequence database of *D. melanogaster*, which was downloaded from the NONCODE website (http://noncode.org) [55].

### Differential gene expression analysis

Clean reads from each developmental stage and tissue were mapped back to our assembled reference transcriptome to estimate the transcript abundance using Bowtie 1.0.0 [56] and RSEM 1.3.0 software [57]. FPKM values for each protein-coding gene were calculated, and genes were defined as expressed when FPKM ≥ 1 in all the biological replicates in this study. FPKM values for each gene were recorded in a data matrix and then imported into edgeR 2.14 software to identify DEGs (fold change > 2^2^) with *P* < 0.001 for the false discovery rate (FDR) [58]. Hierarchical cluster analysis was conducted on the normalized FPKM values of DEGs using the Euclidean distance metric [47]. GO enrichment analysis was conducted using TopGO software using DEGs as input with an adjusted *P* value < 0.001 [59].

### Weighted gene coexpression network analysis

The coexpression network was constructed with WGCNA software [37], and the dataset used in this study consists of 19,023 genes assembled in our transcriptomes of six main different developmental stages and nine different tissues (the testis tissues were removed due to showing high variation compared with other samples). The soft thresholding power (*β*) was first calculated and selected to increase similarity for calculating adjacency. Subsequently, the gene network was constructed; modules were detected using the “blockwiseModules” function implemented in WGCNA software, and the minModuleSize and maxBlockSize were set to be 30 and 20,000, respectively. GO enrichment analysis was performed with TopGO software using the genes in each module as input list [37], [59].

### PCR validation

In this study, real-time quantitative PCR (qRT-PCR) and semi-quantitative PCR were conducted to validate the expression results of the DEGs during larval developmental stages. PCR primer pairs were designed according to our assembled reference transcriptome (Table S15). Three genes, beta-actin (*β-actin*), ubiquitin conjugating enzyme (*Ube*), and ribosomal S27 fusion protein (*S27*) were selected as reference genes for normalization [60]. We conducted qRT-PCR using SYBR Premix Ex Taq (Takara) in a CFX96 real-time PCR system (Bio-Rad, Hercules, CA). The primers with amplification efficiency between 95% and 105% were selected for qRT-PCR. For each selected gene, three technical and three biological replicates were performed. The gene expression level was measured using the 2^−ΔΔCt^ method [61] employing the megalopa in seawater stage as an internal calibration control. One-way ANOVA implemented in SPSS 17.0 was used to determine the statistical significance (*P* < 0.05).

### Phylogenetic analysis

Protein sequences were extracted from the ENSEMBL database (version 84) for *D. melanogaster* and *C. elegans*. Protein sequence data for *P. hawaiensis* were downloaded from the data for the published genome [8]. Protein sequence data for flour beetle (*T. castaneum*), centipede (*Strigamia maritima*), spider mite (*Tetranychus urticae*) and *D. pulex* were downloaded from Ensembl Metazoa (release 31) [52]. For reconstruction of the arthropod phylogenetic tree, single-copy orthologs were identified with orthoMCL (version 2.0.9) [62] from eight selected species, including *D. pulex*, *T. castaneum*, *D. melanogaster*, *S. maritima*, *T. urticae*, *P. hawaiensis*, and *E. sinensis*, as well as *C. elegans* (outgroup). Multiple protein sequence alignments were yielded by T-coffee (version 11) [63] for each orthologous group (OG), and Gblocks (0.91b) software was used to extract conserved well-aligned cores with default parameters [64]. The phylogenetic tree was constructed by MEGA6 based on the amino-acid sequence alignments by means of the neighbor-joining method with 1000 bootstrap replicates [65]. Moreover, we applied RAxML (version 8) to construct the maximum-likelihood phylogenetic tree with the PROTGAMMAJTT substitution model and 100 bootstrap replicates [66].

We further employed MCMCTree in PAML (version 4.8) [67] to estimate the divergence time of the species. The relaxed molecular clock method was employed, using the calibration time as a constraint, including *T. cas*–*D. mel* (~ 275–345 MYA), *S. mar*–*Pancrustacea* (554–625 MYA), and *S. mar*–*T. urt* (568–642 MYA), which were derived from the TimeTree database [68]. The MCMCTree was run for 5,000,000 steps, and the first 5000 samples were burned in.

### Expansion and contraction of gene families

We employed CAFE (version 3.1) software to conduct gene family expansion and contraction analysis using all gene families from orthoMCL [69]. We calculated the probability of each gene family with 10,000 Monte Carlo random samplings and estimated the birth and death rates (λ) of the genes using the maximum-likelihood model. Gene families showing expansion and contraction with *P* < 0.01 were filtered out. Moreover, CAFE can estimate the *P* values of gene families in the extant species that are below the threshold. Thus, branches with low *P* values can be regarded as corresponding to gene family expansions and contractions with accelerated evolution rates.

### Analysis of hemocyanin superfamily genes

Hemocyanin gene families have been believed to play essential roles in physiological processes such as molting, development, respiration, immune and stress response in arthropods [70]. We annotated 25 protein sequences in *E. sinensis* as hemocyanin. We mapped the sequences to other whole-genome sequenced arthropod proteomes using BLASTP (E-value ≤ 1E–6). For phylogenetic analysis, protein sequences from *T. castaneum*, *D. melanogaster*, *Anopheles gambiae*, *E. sinensis*, *P. hawaiensis*, *D. pulex*, *S. maritima*, *Pediculus humanus corporis*, *Stegodyphus mimosarum*, and *Mesobuthus martensii* were aligned using ClustalW, and a JTT+gamma model was used in a maximum-likelihood analysis, employing RAxML to construct a phylogenetic tree [66], [71]. We first removed all partial sequences to construct the initial phylogenetic tree, and then determined the placement of partial sequences one by one based on sequence similarity.

## Data availability

The Transcriptome Shotgun Assembly project and the raw sequencing reads were deposited at GenBank (GenBank: GGQO00000000) and Sequence Read Archive database (SRA: SRX2802626), respectively. The raw sequencing reads were also deposited in the Genome Sequence Archive [72] at the National Genomics Data Center, Beijing Institute of Genomics, Chinese Academy of Sciences / China National Center for Bioinformation (GSA: CRA003690), and are publicly accessible at http://bigd.big.ac.cn/gsa.

## CRediT author statement

**Jun Wang:** Conceptualization, Methodology, Software, Writing - original draft, Funding acquisition. **Xiaowen Chen:** Methodology, Investigation, Visualization. **Funan He:** Methodology, Software, Writing - original draft. **Xiao Song:** Investigation. **Shu Huang:** Investigation. **Wucheng Yue:** Investigation. **Yipei Chen:** Investigation. **Zhixi Su:** Conceptualization, Methodology, Writing - original draft. **Chenghui Wang:** Conceptualization, Supervision, Funding acquisition. All authors read and approved the final manuscript.

## Competing interests

The authors have declared no competing interests.

## Supplementary material

Supplementary Figure S1 **Length distribution of assembled reference transcriptome A.** Length distribution of original transcripts. **B.** Length distribution of filtered transcripts (FPKM > 0.5)

Supplementary Figure S2 **Number of mapping reads and mapping percentage for each developmental stage and****tissue**

Supplementary Table S1 **Number of sequenced raw reads and clean reads for each developmental stage and****tissue**

Supplementary Table S2 **Comparison of assembly and annotation between previous study and this****study**

Supplementary Table S3 **KEGG pathways with numbers of transcripts and enzymes from the transcriptomic analysis of*****Eriocheir sinensis***

Supplementary Table S4 **Prediction result of transcription****factors**

Supplementary Table S5 **Prediction result of non-coding****RNAs**

Supplementary Table S6 **Number of expressed genes and annotated genes in different developmental stages and tissues of*****Eriocheir sinensis***

Supplementary Table S7 **Number of differentially expressed genes (DEGs) in pairwise****comparisons**

Supplementary Table S8 **GO enrichment analysis for upregulated genes in the specific tissues of****adult crabs**

Supplementary Table S9 **Enrichment characterization of functional module****genes**

Supplementary Table S10 **Gene expression information of eight cuticle-related genes identified as differentially expressed in this study by****RNA-seq**

Supplementary Table S11 **1406 one-to-one gene family****list**

Supplementary Table S12 **881 pancrustacea specific gene families881 pancrustacea specific gene****families**

Supplementary Table S13 **GO enrichment analysis of *E. sinensis* specific genes with a *P*****value < 0.05**

Supplementary Table S14 **Significant expansion gene families in*****E. sinensis***

Supplementary Table S15 **Designed primers for qRT-PCR and semi-quantitative PCRDesigned primers for qRT-PCR and semi-quantitative****PCR**
