# Supplementary figures and images for: Global Analysis of Gene Expression Profiles Provides Novel Insights into the Development and Evolution of the Large Crustacean Eriocheir sinensis
Source: Genomics Proteomics Bioinformatics. 2020 Dec 18;18(4):443–54. doi: 10.1016/j.gpb.2019.01.006 (PMC8242267; doi:10.1016/j.gpb.2019.01.006)

**A Original transcript length distribution**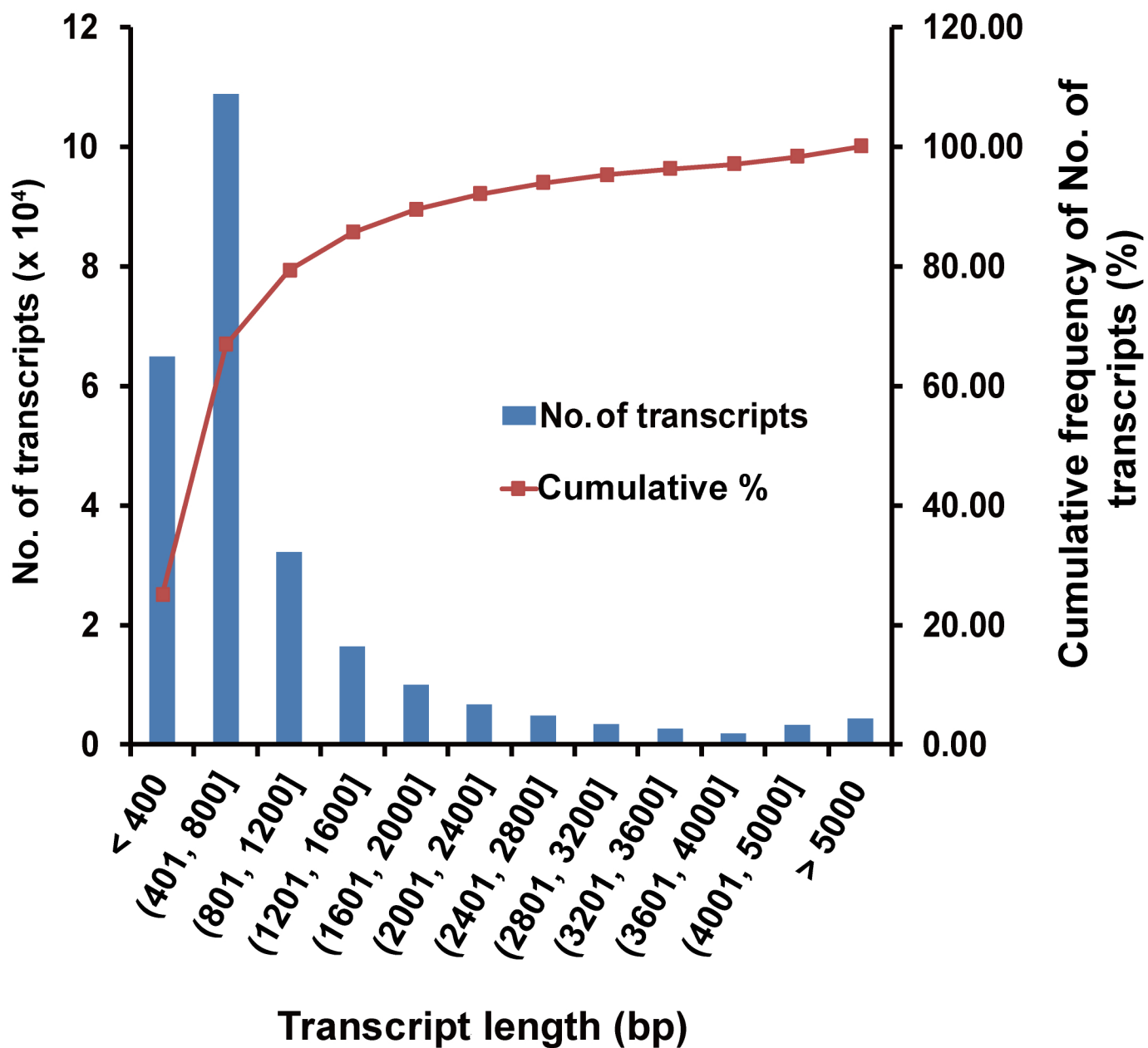**B Filtered transcript length distribution (FPKM > 0.5)**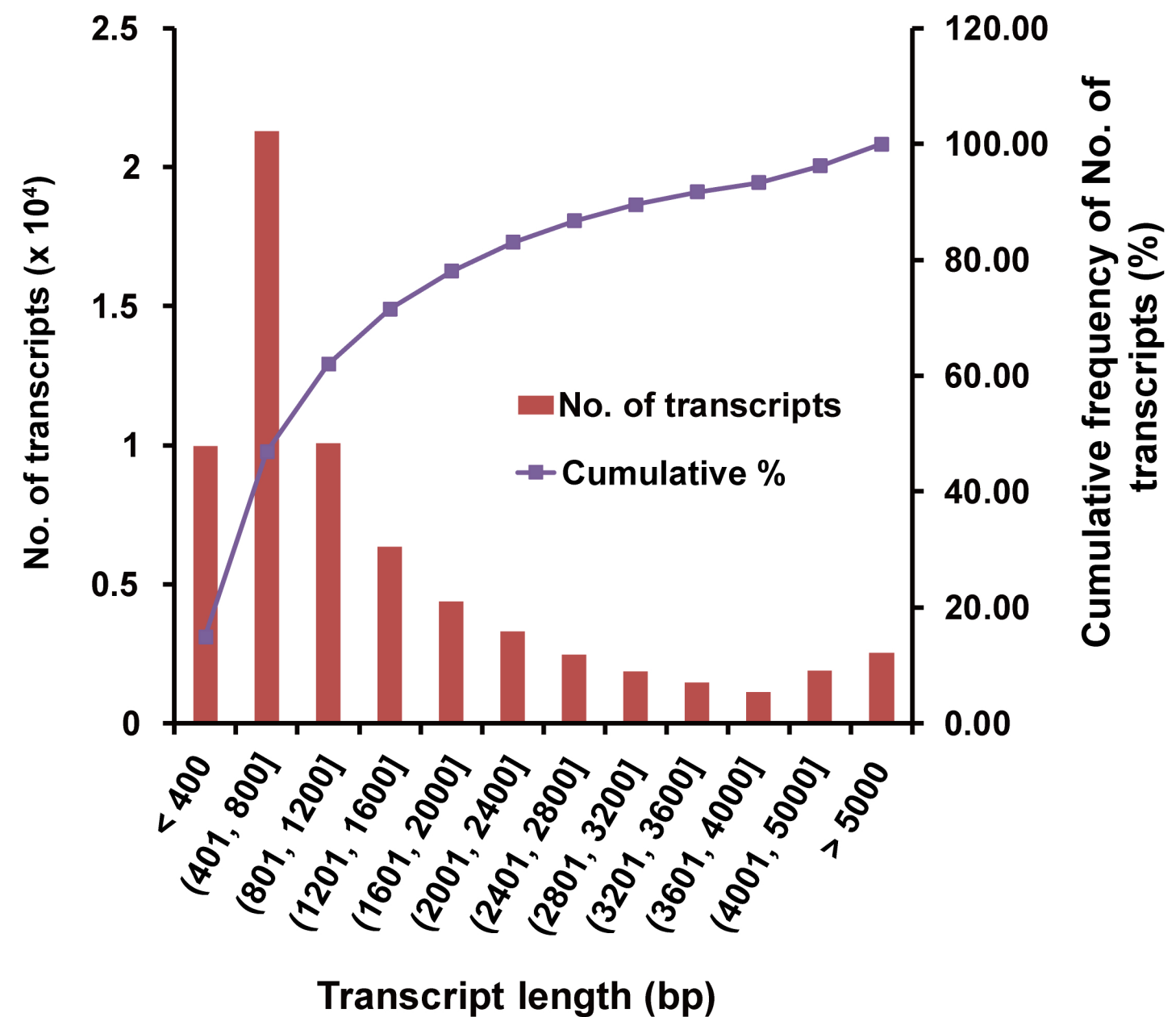

Supplement: Supplementary Figure S1 — Length distribution of assembled reference transcriptome A. Length distribution of original transcripts. B. Length distribution of filtered transcripts (FPKM > 0.5) [file mmc1.pdf]

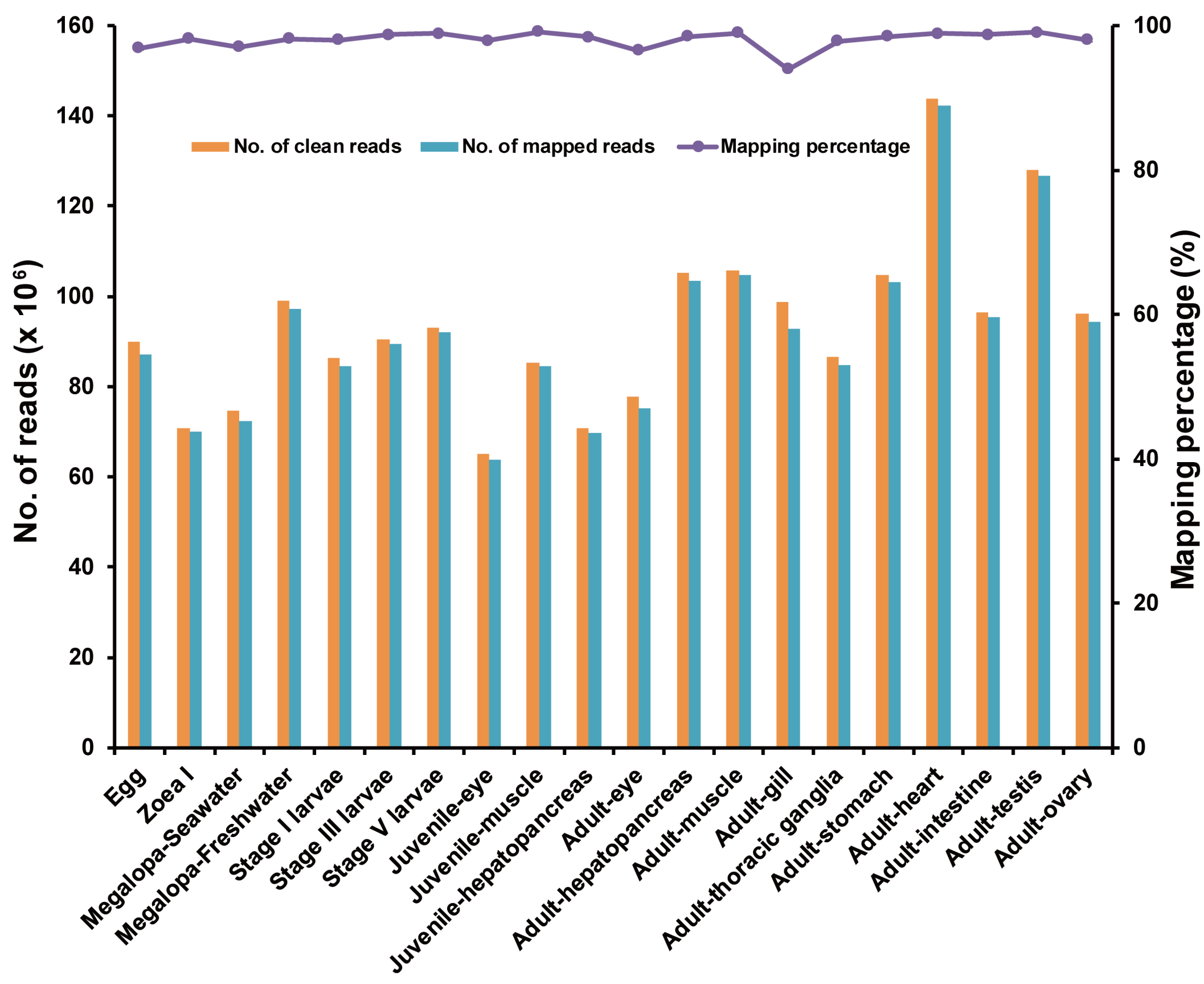

Supplement: Supplementary Figure S2 — Number of mapping reads and mapping percentage for each developmental stage andtissue [file mmc2.pdf]
