# Supplementary material for: Global Analysis of Gene Expression Profiles Provides Novel Insights into the Development and Evolution of the Large Crustacean Eriocheir sinensis
Source: Genomics Proteomics Bioinformatics. 2020 Dec 18;18(4):443–54. doi: 10.1016/j.gpb.2019.01.006 (PMC8242267; doi:10.1016/j.gpb.2019.01.006)
Supplement: Supplementary Table S2 — Comparison of assembly and annotation between previous study and thisstudy [file mmc4.docx]

**Table S2 Comparison of assembly and annotation between previous study and this study.**

|  | **Previous assembly*** | **Our assembly** |
| --- | --- | --- |
| No. of gene models | 7549 | 19,023 |
| N50 length (bp) | 2193 | 2927 |
| Mean length (bp) | 1470 | 1940 |
| No. of annotated gene models | 4990 | 12,015 |

*Note*: * Previous transcriptome assembly was download from Gigascience Database (<http://gigadb.org/dataset/100186>).
